# Alzheimer’s Model Develops Early ADHD Syndrome

**Published:** 2015

**Authors:** Qiang Zhang, Guiping Du, V John, Pankaj Kapahi, Dale E. Bredesen

**Affiliations:** 1 Buck Institute for Research on Aging, 8001 Redwood Blvd., Novato, CA 94945, USA; 2 Department of Neurology, University of California, Los Angeles, CA 90095; 4 Drug Discovery Lab, Department of Neurology, University of California Los Angeles, Los Angeles, CA 90095, USA

**Keywords:** AβPP, Alzheimer’s disease, ADHD, Drosophila

## Abstract

We describe the first invertebrate model of attention deficit hyperactivity disorder (ADHD) that reproduces its major features, including hyperactivity, male predominance, marked exacerbation by simple carbohydrates, reversible response to dextroamphetamine, and a “paradoxical response” to stimulants. This model may offer new insight into ADHD pathogenesis and treatment. Furthermore, these findings are of particular interest in light of the recent epidemiological evidence showing that patients with dementia have a high frequency of antecedent ADHD symptoms.

## Introduction

Alzheimer’s disease (AD) is a major healthcare problem, with over five million patients in the United States, and an annual economic impact of approximately $200 billion. With recent therapeutic trial failures and a rapidly increasing number of cases, improved approaches for high-throughput screening of large compound numbers are needed. To this end, simple models of AD, such as cellular models, invertebrate models, and transgenic mouse models, have been created. However, the invertebrate models described to date display numerous dissimilarities to human AD, such as expression of the relevant gene in muscle or eye rather than brain, and the hyper-expression of the gene, leading to generalized motor reflex deficits [1]. In an effort to create a more relevant *Drosophila* AD model, we utilized the gene switch approach [2], inducibly expressing low levels of human amyloid-β precursor protein (hAβPP) and human β-site AβPP cleaving enzyme 1 (hBACE1). Surprisingly, this expression led to a phenotype highly reminiscent of attention deficit hyperactivity disorder (ADHD), with hyperactivity, male predominance, marked exacerbation by simple carbohydrates, reversible response to dextroamphetamine, and a “paradoxical response” to stimulants, all characteristics of human ADHD. This represents the first invertebrate model of ADHD faithfully reproducing these key features of ADHD.

## Results

**Figure 1:**
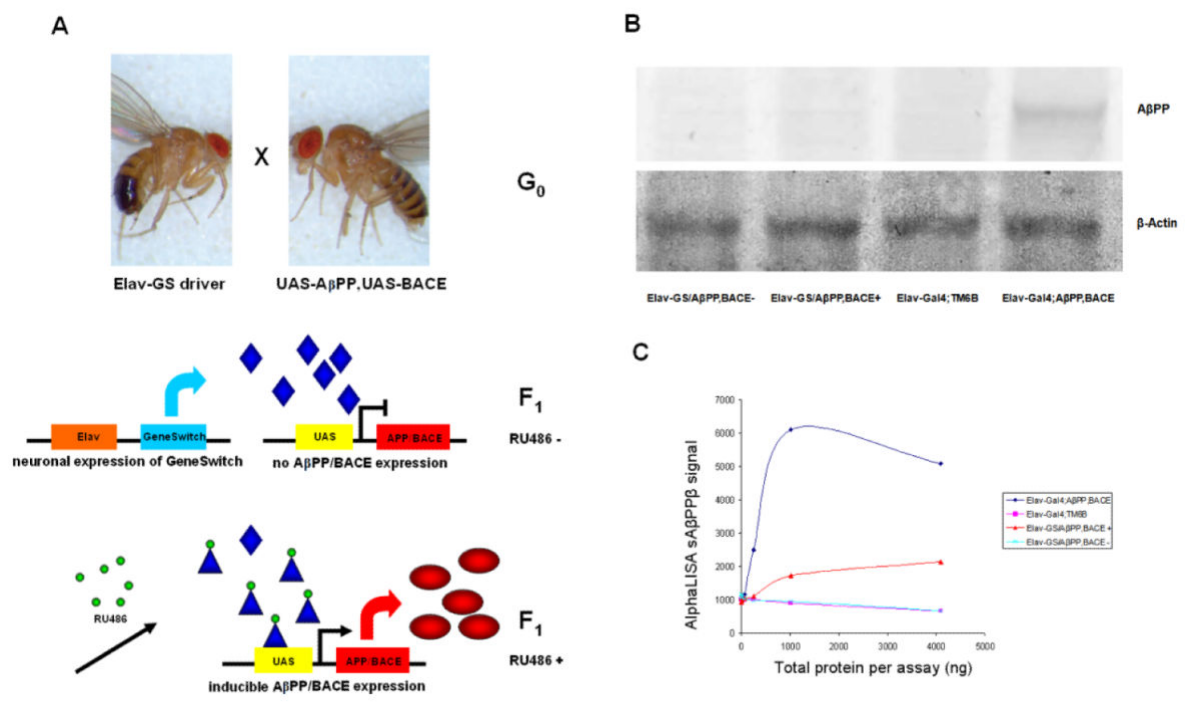
Inducible expression of low levels of hAβPP and hBACE1 in *Drosophila* **A**: RU486-induced expression of hAβPP/hBACE1 in the neuronal tissues of *Drosophila*. **B-C**: Elav-GeneSwitch driver induces low-level hAβPP/hBACE1 expression. High-level expression of hAβPP/hBACE1 was driven by Elav-Gal4. RU486 inducible expression of hAβPP/hBACE1 was driven by Elav-GS. Head lysates were prepared from flies expressing hAβPP/hBACE1, driven by Elav-Gal4 or Elav-GS, and from control lines not expressing hAβPP/hBACE1. These lysates were subjected to Western blot with anti-AβPP antibody and anti-β-actin antibody. For sAβPPβ (cleavage product of hAβPP by hBACE1) assay, lysate samples were subjected to serial dilutions with Alphalisa buffer, and sAβPPβ levels were detected by PerkinElmer AlphaLISA kit.

**Figure 2:**
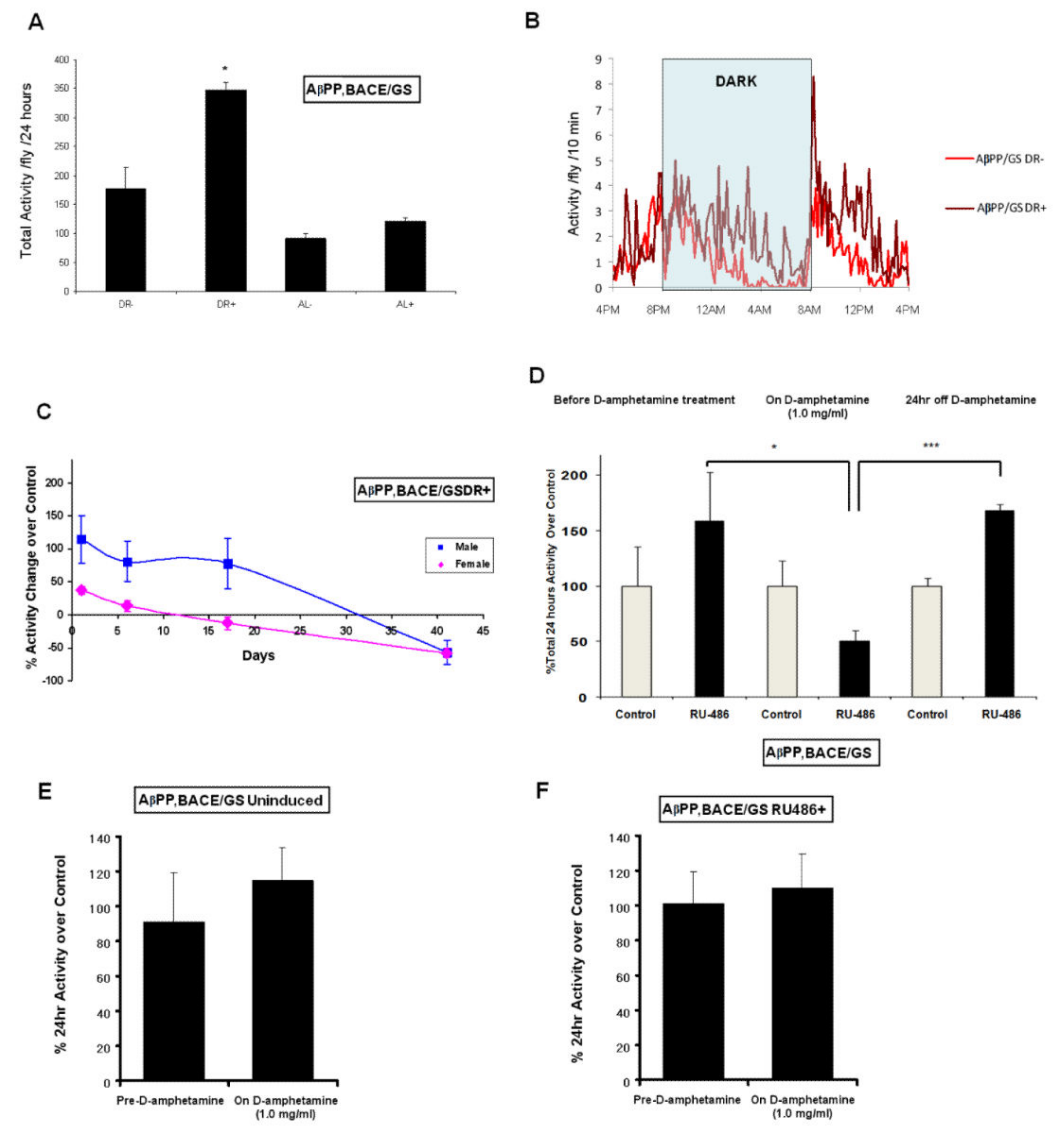
*Drosophila* expressing hAβPP and hBACE1 exhibit hyperactivity with multiple features of ADHD **A-B**: Low-level hAβPP/hBACE1 expression increased the spontaneous activity of flies. **A**: Low-level hAβPP/hBACE1 expression was induced by RU486 (200μM) under the Elav-GS driver. 24 hr spontaneous activity was monitored for flies maintained on DR or AL foods. DR food has sucrose to yeast extract ratio of 10:1, while AL food has a 1:1 sucrose to yeast extract ratio. “+” indicates RU486 induced groups while “−” indicates uninduced groups. Error bar indicates SEM, with n = 3 for each group (* indicates p < 0.05, student t test). **B**: The graph shows averaged activity (three vials per group, with 25 flies in each vial) per 10 min for control and hAβPP/hBACE1 expressing flies. The x-axis represents time (in hr). The activity measurement was started at 4PM. **C**: hAβPP/hBACE1 induced hyperactivity is more prominent in males and disappears as the flies age. 24hr spontaneous activity percent change over control was monitored over 6 weeks. This experiment was repeated three times and similar results were obtained. Error bar indicates SEM. **D**: hAβPP/hBACE1 induced hyperactivity responds to dextroamphetamine treatment. hAβPP/hBACE1 expressing (RU-486 induced) and control (uninduced) flies were treated with 1mg/ml dextroamphetamine. 24hr spontaneous activity was monitored before treatment, during treatment, and 24 hr after treatment. These are male flies fed on DR foods. Error bar indicates SEM, with n = 3 for each group (One way ANOVA, F(2,6)=6.10, p<0.035, pairwise comparisons: * indicates p<0.05, *** indicates p<0.001). **E**: Uninduced flies do not show reduced activity upon dextroamphetamine treatment. Uninduced Elav-GS/UAS-hAβPP,UAS-hBACE1 flies were treated with 1mg/ml dextroamphetamine. The 24hr spontaneous activity of both uninduced dextroamphetamine treatment group flies and the control (uninduced, not treated with dextroamphetamine) flies was monitored before and during treatment. Error bar indicates SEM, with n = 4. **F**: Male flies on AL diet, female flies on DR diet, and female flies on AL diet do not show reduced activity upon dextroamphetamine treatment. hAβPP/hBACE1 expressing flies (Male/AL; Female/DR; Female/AL) were treated with 1mg/ml dextroamphetamine. The 24hr spontaneous activity of both experimental groups and the control (hAβPP/hBACE1 expressing, not treated with dextroamphetamine) groups was monitored before and during treatment. Percentage 24hr activity over control was calculated for each experimental group. Averages of all three groups (Male/AL; Female/DR; Female/AL) were presented. Error bar indicates SEM, with n = 3.

We sought to create an invertebrate model of AD that more closely mimics the human disease in target tissue, mnemonic effects, and response to candidate therapeutics. To create such a *Drosophila* AD model, we used the RU-486-induced Elav-GeneSwitch driver to express low levels of hAβPP and hBACE1 in *Drosophila* (Figure 1). Previous studies have shown that over-expression of hAβPP and hBACE1 leads to severe motor reflex deficits [1]. We therefore employed the *Drosophila* population activity monitors to measure spontaneous activity [3]. Surprisingly, these flies were not found to be hypoactive compared to the uninduced controls, but rather were hyperactive, typically 50-100% more active than the control, uninduced *Drosophila* (Figure 2AB). Interestingly, this effect was more striking on a diet with a high carbohydrate-to-protein ratio (10:1 sucrose to yeast extract) than on a diet with a low carbohydrate-to-protein ratio (1:1 sucrose to yeast extract), a characteristic of human ADHD. Evaluation of the circadian pattern of hyperactivity revealed another characteristic shared by the *Drosophila* model and ADHD patients: nocturnal hyperactivity followed by rapid decline. The characteristic nocturnal pattern in ADHD is wakefulness and activity late into the night followed by the rapid onset of deep sleep (delayed sleep phase syndrome, which is linked genetically with ADHD [4,5]), and the *Drosophila* activity graph was reminiscent of this pattern (Figure 2B). Furthermore, the hyperactivity effect was much more prominent in male flies than in females, and disappeared as the flies aged (Figure 2C). All of these features are highly reminiscent of ADHD, which is more prominent in males, typically maximal in early life, exacerbated markedly by simple carbohydrates, and associated with delayed sleep onset and nocturnal hyperactivity. Two independent strains of *Drosophila* carrying both UAS-hAβPP and UAS-hBACE1 genes were used in these experiments and similar results were obtained with each, indicating that the effects are unlikely to be the result of an insertional event.

We then asked whether a drug used to treat human ADHD could ameliorate the hyperactivity phenotype in *Drosophila*. We fed these flies with food containing 1mg/ml of dextroamphetamine (the major component of Adderall and Dexedrine), and monitored their 24hr spontaneous activity. Just as for human ADHD, treatment with dextroamphetamine rapidly reversed the hyperactivity (Figure 2D). Discontinuation of dextroamphetamine led to a return of hyperactivity (Figure 2D). Interestingly, just as for human ADHD, the effect of dextroamphetamine was “paradoxical,” i.e., the stimulant led to a reduction in activity only in the ADHD-like hyperactive flies, not in the control flies or in any of the other groups that exhibited no, or minimal, hyperactivity (males on low carbohydrate:protein ratio diet, females on low carbohydrate:protein ratio diet, and females on high carbohydrate:protein ratio diet; Figure 2DEF).

**Figure 3:**
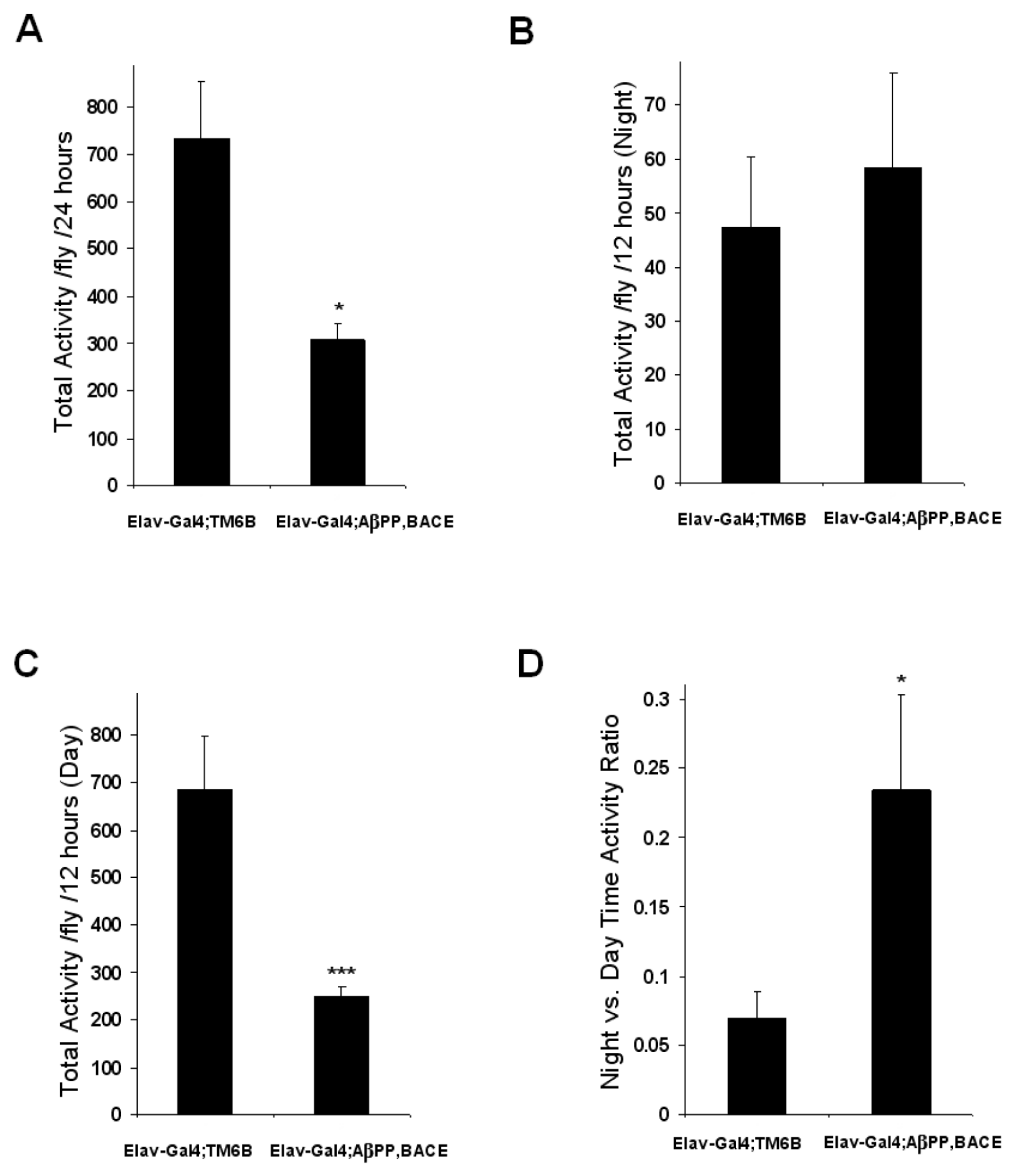
*Drosophila* expressing hAβPP and hBACE1 display disrupted day/night cycle **A-D**: Elav-Gal4 driver was used to induce high levels of hAβPP and hBACE1 expression. **A**: High-level expression of hAβPP and hBACE1 reduced 24 hr total spontaneous activity. The graph shows total spontaneous activity/fly/24hr (five vials per group with 25 flies in each vial) for control and hAβPP/hBACE1 over-expressing flies. **B**: High levels of hAβPP and hBACE1 did not reduce nocturnal (12hr dark cycle) spontaneous activity. **C**: High levels of hAβPP and hBACE1 reduced diurnal (12hr light cycle) spontaneous activity. **D**: High-level expression of hAβPP and hBACE1 significantly increased the ratio of nocturnal to diurnal activity. Error bar indicates SEM, with n = 5 for each group (* indicates p < 0.05, *** indicates p<0.001, student t test).

As noted above, sleep disturbances are common in ADHD, in particular a delay in sleep onset followed by deep sleep [6]. The activity pattern of the hAβPP/hBACE1-expressing flies mimicked this feature of human ADHD, with a delay in nocturnal activity reduction. Moreover, the circadian activity monitor showed that control flies displayed an adaptive monotonic decline in activity following the initial increase associated with light onset and dark onset, while this adaptive decline was delayed by 4-6 hours in the hyperactive flies, suggesting a circadian rhythm abnormality (Figure 2B). When we used the Elav-Gal4 driver to induce higher levels of hAβPP and hBACE1 expression (as compared to the low levels induced using the gene switch), the flies became hypoactive (Figure 3A), probably due to motor reflex deficits as reported previously^1^. However, when we analyzed the diurnal and nocturnal activity separately, we noted that the nocturnal activity was not decreased in hAβPP/hBACE1-expressing flies (Figure 3B); thus the nocturnal:diurnal activity ratio was significantly increased (Figure 3D). These results are compatible with those described above in Figure 2B, again suggesting that the neuronal expression of hAβPP and hBACE1 may affect circadian rhythm regulation, which is another association with ADHD [5].

## Discussion

Thus the inducible expression of modest levels of hAβPP and hBACE1 in *Drosophila* led to a syndrome that reproduces many of the key features of ADHD: (1) a marked increase in overall activity, with males affected more than females; (2) high carbohydrate diets induce hyperactivity; (3) the hyperactivity is mitigated with age; (4) the stimulant dextroamphetamine reduces hyperactivity in a reversible fashion; (5) dextroamphetamine does not reduce activity in the non-ADHD model groups; (6) the nocturnal pattern of activity features a delay in activity reduction, with a rapid loss of activity late in the 12hr night/dark cycle. This represents the first invertebrate model of ADHD that displays these characteristics of ADHD. Since the underlying mechanisms for these features of ADHD are not well understood, the existence of a genetically tractable model that displays all of these key features of ADHD should provide a valuable tool to identify candidate mechanisms, as well as a simple model for pharmacological screens.

It is noteworthy that patients with dementia have recently been described as displaying an increase in antecedent symptoms of ADHD [7]. Interestingly, this increase was observed in patients with dementia with Lewy bodies (DLB), which exhibits features of both Alzheimer’s disease and Parkinson’s disease. DLB demonstrates an increased amyloid load in over 80% of cases [8], which has led to the suggestion that DLB therapy should include anti-amyloid approaches. Furthermore, therapy with cholinesterase inhibitors, shown to have a modest effect on AD, may yield a similar or even greater therapeutic benefit in DLB [9], offering another parallel between these two conditions.

Pathologically, DLB features both amyloid plaques with AβPP fragments and Lewy bodies with α-synuclein and AβPP [10]. Moreover, similar to ADHD, both DLB and AD patients exhibit sleep disturbances [11]. It should be added that, although we did not evaluate attention span in the current study, van Swinderen and Brembs have reported that the *Drosophila* memory mutant, *radish*, in addition to its memory defect, displays an apparent attention deficit, responsive to methylphenidate [12].

Why the inducible expression of hAβPP and hBACE1 leads to a syndrome in *Drosophila* that reproduces so many of the key features of ADHD is not yet explained; however, it should be noted that mouse models of AD also display hyperactivity [13], and patients with Down syndrome may also exhibit ADHD-like symptoms prior to the development of dementia [14]. We speculate that reduced monoaminergic signaling, similar to what has been described in ADHD, occurs in the *Drosophila* model, in the latter case due to reduced connectivity caused by the expression of hAβPP and hBACE1. Whatever the mechanism(s), however, these findings offer a simple model to dissect ADHD mechanisms, as well as a rapid and sensitive *in vivo* pre-rodent/post-cell screen for drug efficacy to reverse the ADHD phenotype.

## Methods

### RIGOR guidelines for translational research

This study adheres to current RIGOR guidelines of for translational research [15,16], with appropriate control groups and statistical analysis as detailed below.

### Fly strains and Fly husbandry

Flies were developed on standard lab food (Caltech food recipe) at 25°C, and for spontaneous activity measurement the adults were transferred within 0-4 days of eclosion to yeast extract (YE) diet (variable concentrations of YE) as described previously [17]. The AL (ad libitum) diet contained 5% yeast extract and 5% sucrose while the DR (dietary restriction) diet had 0.5% yeast extract and 5% sucrose. Males carrying the RU-486 inducible Elav-GS driver (a kind gift from Dr. Haig Keshishian [2]) or the Elav-Gal4 driver (BL#458, BL# refers to Bloomington Stock Center stock number) were crossed to virgin females carrying the UAS-AβPP and UAS-hBACE1 genes (two lines, one from Dr. Daniel Marenda and Dr. Rita Reifegerste [1], the other line from Bloomington Stock Center (BL#33797)).

High-level expression of hAβPP/hBACE1 was achieved by crossing Elav-Gal4/y;+/+;+/+ males and +/+;+/+;TM6B/UAS-hAβPP,UAS-hBACE1 virgin females. RU486 inducible expression of hAβPP/hBACE1 was achieved by crossing +/y;+/+;Elav-GS/Elav-GS males and +/+;+/+;TM6B/UAS-hAβPP,UAS-hBACE virgin females (Fig1).

Adults from the progeny were then transferred to food with varying concentrations of YE in the absence or presence of 200μM RU-486 and were maintained at 25°C for spontaneous activity measurements. RU-486 was obtained from Sigma-Aldrich (Cat #: M8046, Purity: >98%). RU-486 induction was started 0-4 days after eclosion, and spontaneous activity measurement were performed 24hours after initiation of RU-486 induction. Briefly, after 24hr of RU-486 induction, flies were transferred into vials with foods containing RU486 at 9AM in the morning. The vials were loaded onto the monitor and the flies were allowed to feed and settle down. 24hr activity recording was started at 4PM. For D-amphetamine exposure, flies induced with RU486 for 72 hours were transferred to food containing D-amphetamine and RU486 at 9AM in the morning, and again, 24hr activity recording was started at 4PM. Appropriate control groups not induced with RU486 were monitored at the same time (fed on food containing same concentration of Ethanol at 0.2%). Ethanol was used as solvent to dissolve and evenly distribute RU486 (100mM stock solution was diluted 1:500 in food). Control groups were fed on foods containing the same amount of vehicle ethanol, but not RU486.

### Pharmacology

Dextroamphetamine hemisulfate (D-Amphetamine hemisulfate salt, Sigma-Aldrich,St. Louis, MO, cat #A5880) was dissolved freshly in water and mixed with food at 1mg/ml. Flies were moved into vials with drug-laced food at 9AM, allowed to eat for 7hr and monitored for 24hr spontaneous activity, starting at 4PM.

### Spontaneous activity measurements

For measurement of spontaneous activity we used *Drosophila* activity monitors (Trikinetics Inc., Waltham, MA). The instrument measures the movement of flies in the vertical direction and at three equidistant points over the length of a vial (approximately 2 cms, 5 cms and 8 cms above the food surface). For a 24 hr measurement, the flies were first transferred to fresh food in the morning at 9:00 am and then moved to the counters at 4:00 pm for measurements for the next 24 hr. The data were collected, pooled, and recorded every 10 minutes.

### Western Blot and AlphaLisa

Head lysates were prepared from Elav-Gal4/+;+/+;+/UAS-hAβPP,UAS-hBACE1 females, Elav-Gal4/+;+/+;+/TM6B females, RU486 induced +/+;+/+;Elav-GS/UAS-hAβPP,UAS-hBACE1 females, and uninduced +/+;+/+;Elav-GS/UAS-hAβPP,UAS-hBACE1 females. These lysates were subjected to Western blot with anti-AβPP antibody and anti-β-actin antibody. Briefly, 100 fly heads were collected from respective genotypes and immediately lysed in RIPA buffer (50 mM Tris, 150 mM NaCl, 1% SDS, 1% NP-40, 0.5% deoxycholate, pH 7.5) containing a cocktail of protease inhibitors (Roche, complete mini). These lysates were stored at −80°C. The protein concentration of these fly head lysates was determined using the BCA Protein Assay Kit (Pierce, Inc.). According to the protein concentrations, samples for Western blots were prepared using the NuPAGE LDS sample buffer (Invitrogen, Inc.) containing 50mM DTT (Sigma-Aldrich). Equal amounts of protein were loaded into each well of NuPAGE 4–12% Bis Tris Gel. From the gel the proteins were transferred onto PVDF (Immobilon P) membrane (Millipore). Blots were probed with anti-AβPP (5A3/1G7, a kind gift from Dr. Edward Koo) and anti-β-actin (Cell Signaling) antibodies.

For sAβPPβ (cleavage product of hAβPP by hBACE1) assay, lysate samples were subjected to serial dilutions with AlphaLISA buffer, and sAβPPβ levels were detected by PerkinElmer AlphaLISA kit (PerkinElmer, Waltham, MA), and measured using a PE-Enspire 96-well plate reader.

### Statistics

Raw data were statistically analyzed using one-way ANOVA (GraphPad Prism software; San Diego, CA), followed by between-group comparisons using the Newman-Keuls test. P < 0.05 was considered statistically significant.

## Abbreviations

AβPP: amyloid-β precursor protein
BACE: β-site AβPP cleaving enzyme
ADHD: attention deficit hyperactivity disorder
